# Surgically modifiable factors measured by computer-navigation together with patient-specific factors predict knee society score after total knee arthroplasty

**DOI:** 10.1186/s12891-016-0929-7

**Published:** 2016-02-13

**Authors:** Frank Lampe, Franziska Fiedler, Carlos J. Marques, Anusch Sufi-Siavach, Georg Matziolis

**Affiliations:** Research Center of the Department of Orthopedics and Joint Replacement at the Schoen Klinik Hamburg Eilbek, Dehnhaide 120, D-22081 Hamburg, Germany; Department of Orthopedics and Joint Replacement at the Schoen Klinik Hamburg Eilbek, Dehnhaide 120, D-22081 Hamburg, Germany; Faculty of Life Sciences at the Hamburg University of Applied Sciences, Lohbrügger Kirchstraße 65, D-21033 Hamburg, Germany; Orthopaedic Department, Jena University Hospital, Campus Eisenberg, Klosterlausnitzer Straße 81, D-07607 Eisenberg, Germany

**Keywords:** Total knee replacement, Computer-assisted surgery, Prognosis, Outcome assessment

## Abstract

**Background:**

The purpose was to investigate whether patient-specific factors (PSF) and surgically modifiable factors (SMF), measured by means of a computer-assisted navigation system, can predict the Knee Society Scores (KSS) after total knee arthroplasty (TKA).

**Methods:**

Data from 99 patients collected during a randomized clinical trial were used for this secondary data analysis. The KSS scores of the patients were measured preoperatively and at 4-years follow-up. Multiple regression analyses were performed to investigate which combination of variables would be the best to predict the 4-years KSS scores.

**Results:**

When considering SMF alone the combination of four of them significantly predicted the 4-years KSS-F score (*p* = 0.009), explaining 18 % of its variation. When considering only PSF the combination of age and body weight significantly predicted the 4-years KSS-F (*p* = 0.008), explaining 11 % of its variation. When considering both groups of predictors simultaneously the combination of three PSF and two SMF significantly predicted the 4-years KSS-F (*p* = 0.007), explaining 20 % of its variation.

**Conclusions:**

Younger age, better preoperative KSS-F scores and lower BMI before surgery, a positive tibial component slope and small changes in femoral offset were predictors of better KSS-F scores at 4-years.

## Background

Total knee arthroplasty (TKA) is an effective and cost-efficient [1–3] intervention for end-stage knee osteoarthritis (OA). The outcomes by which the success of TKA can be measured are different. Pain relief and the restoration of functional activities have been reported as the main outcomes after primary TKA [4]. Other authors have focused on aspects influencing health-related quality of life (HRQL) [5–9] or patient satisfaction [10–12].

In the field of outcome-research there is an increasing interest in understanding the factors that influence and may predict the results after TKA. In this context there are patient-specific factors (PSF) and surgically modifiable factors (SMF) that can be considered as potential predictors of outcomes. Examples of PSF are the preoperative range of movement (ROM) [13, 14], body mass index (BMI) [15] and the presence or absence of co-morbidities [16, 17]. Under SMF one could consider, among others, the type of prosthesis used [18, 19], changes in posterior tibial slope (PTS) [14] and changes in posterior condylar offset (PCO) [14]. In the past these factors could only be assessed by radiographs, with the well-known limitations of radiometric morphometry. Using computer-navigation systems a large variety of biomechanical parameters can be monitored and saved intra-operatively. Several meta-analyses have shown that navigated TKA can reduce the number of radiographic outliers compared with traditional techniques, leading to significant improvements in prosthesis alignment and positioning [20–24].

From a clinician perspective, clinical scores are of great importance as endpoints because they are easy to apply. Their use is widespread, allowing comparisons between different populations. The knee society score (KSS) [25] is a validated rating system generally used to evaluate both the knee function and patient functional ability before and after TKA. With its KSS-Knee and KSS-Function sub-scores, it combines an objective physician-derived with a patient-subjective component. Pain relief, function and patient satisfaction can be evaluated.

The purpose of this study was to investigate the predictive value of PSF and SMF measured by means of a computer-assisted navigation system on the KSS ratings. It was hypothesized that a combination of PSF and SMF could explain a proportion of KSS variability at 4-years after surgery.

## Methods

### Subjects

This study is a secondary analysis of data obtained during a prospective randomized clinical trial designed to investigate the effects of mobile bearing (MB) vs. fixed-bearing (FB) TKA implants on clinical scores [26].

Ninety-nine patients (100 knees) scheduled for primary bicondylar, posterior cruciate retaining TKA at the Schoen Klinik Hamburg Eilbek were informed about the study and agreed to participate.

All patients met the following inclusion criteria: clinical and radiological signs of osteoarthritis of the knee with failed non-operative treatment; no indication for a uni-compartmental implant or joint-preserving osteotomies; age ranging from 40 to 90 years; American society of anaesthesiologists pre-operative classification grade 1–3; no deformity larger than 20° varus or 15° valgus; no previous bone surgery to the index knee; no previous total joint replacement at the index leg; no post-operative infection of the index knee or thrombosis within the follow-up period.

The patients were randomly assigned into one of the groups. At baseline there were 52 patients in the FB and 48 in the MB group. At 4 years follow-up there was a loss of 7 patients (13.5 %) in the FB group (2 died with no relation to TKA; 5 did not attend the 4 years follow-up) and 6 patients (12.5 %) in the MB group (1 septic implant exchange before 12 months; 3 did not attend the 1 year follow-up; 1 died with no relation to TKA).

The mean age of the patients by entrance in the study was 69.1 ± 7.8 years. The distribution of female and male patients in the sample was 73 and 27, respectively. There was no significant difference between the mean ages of male and female patients (*p* = 0.7). The significant differences found when comparing the mean body weight (*p* = 0.02) and mean body height (*p* < 0.001) of both genders had no consequences in terms of mean BMI differences (*p* = 0.2). For further demographic data see Table 1.

**Table 1:** Demographic data of the sample by the time of entry in the study

Variables	All	Female	Male	Mean Diff. (*p*-value) [95 % CI]
Number of Patients	*n* = 100	*n* = 73	*n* = 27	
Age (years)	69.1 ± 7.8	69.3 ± 7.9	68.7 ± 7.4	0.5 (*p* = 0.7) [-4.0 to 2.9]
Body Weight (Kg)	82.6 ± 15.7	80.5 ± 15.5	88.2 ± 14.8	7.7 (*p* = 0.02) [0.8 to 14.6] ^(*)^
Body Height (cm)	167.1 ± 8.4	163.8 ± 6.4	176.0 ± 6.4	12.1 (*p* < 0.001) [9.2 to 15.0] ^(*)^
BMI (Kg/m^2^)	29.5 ± 5.5	29.9 ± 5.7	28.5 ± 5.0	1.3 (*p* = 0.2) [-3.8 to 1.1]

Values are mean ± SD; ^(*)^ = Significant difference

Since there were no significant differences between the KSS scores in the fixed- and mobile-bearing groups at any measurement time, and no significant differences when comparing the ROM of both groups across the follow-up assessments [26], all patients were pooled into one group for the purpose of this secondary analysis of the data.

Before participating all patients were required to read and sign an informed consent form, in which their permission to anonymously save the navigation data registered during surgery for the purpose of this secondary data analysis was also requested. The medical Ethics Commission of the Federal State of Hamburg approved the research proposal (File #2226). The trial was registered under ClinicalTrial.gov (NCT00822640).

### Materials

The computer-assisted navigation system OrthoPilot TKA version 4.2 (BBraun Aesculap, Tuttlingen, Germany) was used to perform all surgeries. The system is based on intra-operatively acquired data. An infrared camera tracks infrared diodes, which have been previously fixed on the femoral and tibia bones, on a hand-held pointer and on the cutting blocks [27]. The optical tracking system used by OrthoPilot is the hybrid Polaris Spectra® (Northern Digital Inc., Waterloo, Ontario, Canada). According to the supplier, the accuracy of the system is 0.25 mm RMS when applying the pyramid measurement volume method [28, 29].

The navigation system defines the mechanical axis of the leg. With navigation-adapted instruments the surgeon performs the femoral and tibial resection cuts under real-time control of the navigation parameters, allowing leg alignment corrections and soft-tissue balance. The reliability of leg alignment when using this system, as well as the reliability of the navigation guided gap technique were tested in the past in experimental settings [30, 31].

The extension and flexion gaps were measured after tibial cutting by the navigation system after introducing a device (Laminar spreader, BBraun Aesculap, Tutlingen, Germany) allowing independent tensioning of the medial and lateral joint gaps. Based on these measurements the femoral component size and position were planned, aiming to minimize gap inequalities and asymmetries as far as possible (tibia first technique). The measurement and calculation of the navigation variables was made on the bases of the resected bone surfaces. No additional measurements were performed after the implantation of the implant components. Detailed information on the surgical procedure and workflow when using the OrthoPilot system were published previously [32].

### Measurements

For the present purpose preoperative and follow-up data acquired at 4- years post-surgery by a trained physician were used.

All surgeries were performed by one of two senior surgeons. Navigation data were recorded during surgery. Some navigation variables were calculated post-surgery based on the data obtained intra-operatively.

The fifteen SMF used in this study are explained in Table 2. Furthermore, the following PSF were used: patient’s age by the time of surgery, body weight, body height, BMI, preoperative maximal knee flexion (Pre-OP MKF) and preoperative KSS scores (Pre-OP KSS).

**Table 2:** Definition of the surgically modifiable factors (SMF)

Name	Abbreviation	Definition	Values
Femoral Joint Line Change max.	FJLCmax	Femoral joint line change from the most prominent condyle	Millimeter
Femoral Joint Line Change min.	FJLCmin	Femoral joint line change from the less prominent condyle	Millimeter
Femoral Component Slope	FS	Femoral component angle in the sagittal plane	Degrees
Tibial Joint Line Change	TJLC	Tibial joint line change from the unworn compartment	Millimeter
Tibial Component Slope	TCS	Tibial component angle in the sagittal plane	Degrees
Extension Gap Size Medial	EGSmed	Medial gap between the femoral and tibial components in extension	Millimeter
Extension Gap Size Lateral	EGSlat	Lateral gap between the femoral and tibial components in extension	Millimeter
Extension Gap Difference Medial-Lateral	EGDM-L	Difference between EGSmed and EGSlat (EG symmetry)	Millimeter
Flexion Gap Size Medial	FGSmed	Medial gap between the femoral and tibial components in 90° flexion	Millimeter
Flexion Gap Size Lateral	FGSlat	Lateral gap between the femoral and tibial components in 90° flexion	Millimeter
Flexion Gap Difference Medial-Lateral	FGDM-L	Difference between FGSmed and FGSlat (FG symmetry)	Millimeter
Flexion Extension Gap Difference	FEGD	Difference between the mean EGS and the mean FGS (Gap equality)	Millimeter
Femoral Offset Changes Medial	FOCmed	Difference between the medial FO before and after femoral component implantation	Millimeter
Femoral Offset Changes Lateral	FOClat	Difference between the lateral FO before and after femoral component implantation	Millimeter
Femoral Rotation	FR	Femoral rotation relative to the posterior condyle line	Degrees

### Statistical analysis

Mean and standard deviation were used to describe the data, since all predictor variables are continuous. Normal distribution of the data was confirmed with the Kolmogorov-Smirnov test. Demographic data comparisons between male and female patients in the sample were performed on baseline data with a *t*-test for independent samples (Table 2).

Multiple regression analyses to estimate the contribution of different independent variables (SMF and PSF) to the explanation of 4-years KSS (dependent variable) were carried out in three phases. In the first phase Pearson’s correlation was used to assess the univariate association between each independent variable (potential predictors) and the 4-years KSS scores. A *p*-value ≤ 0.20 was accepted as the level of significance to ensure that potentially relevant independent variables were not excluded at this stage as recommended in the literature [33]. In the second phase all independent variables that were significantly associated with the 4-years KSS-F score were entered into two backward multiple regression models. A stepping method criterion with a probability of F to remove ≥ 0.10 was used. The first model included only SMF and the second model included only PSP. Finally, in the third phase, a final model was generated including selected SMF and PSF. The selection of the independent variables for the third model was made based on the results of the two previous procedures, on the literature and on surgeons empirical knowledge. Since the unstandardized regression coefficients (B) communicate the direction (positive or inverse) and the weighting of the independent variable (predictor) relative to the other independent variables in explaining the variation of the dependent variable, only predictors with a unstandardized coefficient, which could be explained according to the existing literature were included in the final model.

The three models met the assumptions of multiple regressions in terms of linearity, homoscedasticity, normality, independence and non-multicollinearity.

All statistical tests were carried out with the use of the IBM SPSS software version 21 for Mac. For all statistical tests the 0.05 level of probability was accepted as the criterion for statistical significance.

## Results

The correlation coefficients between the 15 SMF and the 4-years KSS-Function scores are presented in Table 3. Only six had a significant linear relationship with the 4-years KSS-F (Table 3). When considering only the SMF as potential predictors, the prediction model generated contained four of the six variables (FJLCmin, TCS, FGDM-L, FOCmed) and was reached in three steps (Table 4). The model was statistically significant, F _(4, 95)_ = 3.7, *p* = 0.009, and accounted for approximately 18 % of the variance of the 4-year KSS-F score (*R*^*2*^ = 0.18, Adj. *R*^*2*^ = 0.13). Three of the four predictors added statistically significantly to the prediction.

**Table 3:** Correlations between surgically modifiable factors (SMF) and 4-years KSS-F scores

Potential Predictor (Surgically modifiable variables)	**KSS-F**	FJLCmax	FJLCmin	FS	TJLC	TCS	EGSmed	EGSlat	EGDM-L	FGSmed	FGSlat	FGDM-L	FEGD	FOCmed	FOClat	FR
**Femoral Joint Line Change max** (FJLCmax)	**.25** ^*^															
**Femoral Joint Line Change min** (FJLCmin)	**.29** ^*^	.52^*^														
Femoral Component Slope (FS)	-.04	-.06	-.23^*^													
Tibial Joint Line Change (TJLC)	-.02	-.37^*^	-.16	-.14												
**Tibial Component Slope** (TCS)	**.22** ^*^	.07	-.02	.30^*^	-.00											
Extension Gap Size Medial (EGSmed)	.05	.07	-.00	.00	-.24^*^	-.02										
Extension Gap Size Lateral (EGSlat)	.03	.32^*^	-.17	.17	-.33^*^	.14	.20									
Extension Gap Difference Med-Lat (EGDM-L)	.08	-.04	.13	-.02	.00	.03	-.22^*^	.31^*^								
Flexion Gap Size Medial (FGSmed)	.05	-.29^*^	-.15	.08	.17	-.09	-.07	-.16	-.11							
Flexion Gap Size Lateral (FGSlat)	-.04	-.30^*^	-.02	.08	.13	-.12	-.08	-.18	-.03	.71^*^						
**Flexion Gap Difference Med-Lat** (FGDM-L)	**.16** ^**^	.05	.06	-.01	.09	.04	.00	.05	.08	.05	-.41^*^					
Flexion-Extension Gap Difference (FEGD)	-.02	-.36^*^	-.02	-.03	.37^*^	-.07	-.34^*^	-.53^*^	-.02	.54^*^	.62^*^	-.18				
**Femoral Offset Changes Medial** (FOCmed)	**-.17** ^**^	.25^*^	.11	-.00	-.36^*^	.05	.26^*^	.17	-.02	-.29^*^	-.54^*^	.31^*^	-.45^*^			
**Femoral Offset Changes Lateral** (FOClat)	**-.14** ^**^	.33^*^	.12	-.00	-.33^*^	.07	.25^*^	.23^*^	.03	-.36^*^	-.50^*^	.30^*^	-.45^*^	.90^*^		
Femoral Rotation (FR)	.05	.16	.07	-.03	.16	-.01	-.08	.05	.09	-.05	.18	-.04	.08	-.33^*^	.08	
Mean ± SD	85.2 ± 15.6	1.5 ± 1.9	0.7 ± 2.3	0.1 ± 1.2	3.0 ± 1.7	3.9 ± 1.1	0.6 ± 1.2	1.7 ± 2.1	1.9 ± 1.4	3.1 ± 1.4	2.8 ± 1.6	0.7 ± 0.9	2.2 ± 1.6	2.6 ± 2.7	0.3 ± 2.5	3.4 ± 1.3

^(*)^ *p* < .05; ^(**)^ *p* < .20 (Statistically significant linear relationship to 4-Y KSS-F in bold)

**Table 4:** Multiple regression models for 4-years KSS-F scores

	Dependent variable	Step	Predictors included	Predictors excluded	R^2^	Adj. R^2^	F	p*	B	p**
1^st^ Model (only SMF)	4-years KSS-F	1	All six		0.20	0.12	2.6	**0.02**		
		2		FOClat	0.20	0.13	3.1	**0.01**		
		3	FJLCmin	FJLCmax	0.18	0.13	3.7	**0.009**	1.7	**0.02**
			TCS						2.8	**0.04**
			FGDM-L						3.7	**0.03**
			FOCmed						-1.3	0.05
2^nd^ Model (only PSF)	4-years KSS-F	1	All four		0.11	0.07	11.4	**0.04**		
		2		BMI	0.11	0.08	3.5	**0.01**		
		3	Age	Pre-OP KSS-F	0.11	0.08	5.1	**0.008**	-0.2	**0.04**
			Body Weight						-0.6	**0.03**
3^rd^ Model (combination of PSF and SMF)	4-years KSS-F	1	BMI		0.20	0.14	3.4	**0.007**	-0.7	**0.02**
			Age						-0.7	**0.001**
			Pre-OP KSS-F						0.04	0.5
			TCS						2.2	0.1
			FOCmed						-0.7	0.2

p* = Statistical significance of the model; B = Unstandardized Coefficients; p** = Statistical significance of the predictors included in the final model

Significant *p*-values in bold; *SMF* Surgically modifiable factors, *PSF* Patient-specific factors

The correlation coefficients between PSF and the 4-years KSS-F scores are shown in Table 5. Four of the six PSF (body weight, BMI, age and Pre-OP KSS-F) had a significant linear relationship with the KSS-F score (Table 5). The prediction model generated contained two predictors (age and body weight) and was reached in three steps (Table 4). The model was statistically significant, F _(2, 97)_ = 5.1, *p* = 0.008, and explained approximately 11 % of the variance of 4-years KSS-F (*R*^*2*^ = .11, Adj. *R*^*2*^ = .08). Both predictors added statistically significantly to the prediction.

**Table 5:** Correlations between patient-specific factors (PSF) and 4-years KSS-F scores

Potential	**4-years**	Body	Body	BMI	Age	Pre-OP	Pre-OP
Predictors	**KSS-F**	Weight	Height			KSS-F	MKF
**Body Weight**	**-.11** ^**^						
Body Height	-.04	.31^*^					
**BMI**	**-.09** ^**^	.86^*^	-.20^*^				
**Age**	**-.25** ^*^	-.35^*^	-.13	-.31^*^			
**Pre-OP KSS-F**	**.08** ^**^	-.09	.04	-.12	-.01		
Pre-OP MKF	.03	-.00	.19^*^	-.09	-.12	.08	
Mean ± SD	85.0 ± 15.1	83.4 ± 15.9	167.1 ± 8.5	29.9 ± 5.6	68.9 ± 7.9	48.5 ± 19.8	110.0 ± 14.2

^(*)^ *p* < .05; ^(**)^ p< .20 ; Statistically significant linear relationship to 4-Y KSS-F score in bold. *MKF* maximal knee flexion

Finally, the combination of BMI, age, Pre-OP KSS-F, TCS and FOCmed significantly predicted 4-years KSS-F, F _(5, 94)_ = 3.7, *p* = 0.007, and accounted for approximately 20 % of the variance of 4-years KSS-F (*R*^*2*^ = .20, Adj. *R*^*2*^ = .14). Lower BMI, younger age and higher KSS-F scores by the time of surgery, together with a positive (posterior) TCS and a good reconstruction (small changes) in FOCmed led to better KSS-F scores at 4-years. Only two of the five predictors added statistically significantly to the prediction (Table 4).

The correlation coefficients between SMF, PSF and the 4-years KSS-Knee scores are presented in Table 6. There were no significant correlations between the SMF and the KSS-Knee score at 4-years for a *p* < 0.05. As described above, a *p*-value ≤ 0.20 was accepted as the level of significance to ensure that potentially relevant independent variables were not excluded at this stage. Eight potential predictors (see Table 6) were selected and entered into a backward multiple regression model. The models created were not statistically significant. The same procedure was repeated for PSF. Again, no statistically significant models were found. A negative negligible relationship between KSS-K preoperatively and KSS-K at 4-years was statistically significant (*p* < 0.05).

**Table 6:** Correlations between SMF, PSF and 4-years KSS-Knee scores

Potential predictors surgically modifiable variables	KSS-K
**Femoral Joint Line Change max** (FJLCmax)	.18^**^
**Femoral Joint Line Change min** (FJLCmin)	.12^**^
Femoral Component Slope (FS)	.06
Tibial Joint Line Change (TJLC)	-.06
**Tibial Component Slope** (TCS)	.18^**^
Extension Gap Size Medial (EGSmed)	.06
**Extension Gap Size Lateral** (EGSlat)	.16^**^
**Extension Gap Difference Med-Lat** (EGDM-L)	.21^**^
Flexion Gap Size Medial (FGSmed)	.01
Flexion Gap Size Lateral (FGSlat)	-.09
Flexion Gap Difference Med-Lat (FGDM-L)	-.09
**Flexion-Extension Gap Difference** (FEGD)	-.16^**^
**Femoral Offset Changes Medial** (FOCmed)	-.11^**^
Femoral Offset Changes Lateral (FOClat)	-.07
**Femoral Rotation** (FR)	.11^**^
**Patient-specific factors**	
Body weight	-.11
**Body height**	-.04^**^
**BMI**	-.09^**^
Age	.00
**KSS-K Preoperatively**	-.17^*^
Maximal knee Flexion (MKF) Preoperatively	-.04
Mean (KSS-K) ± SD	87.2 ± 10.3

Potential predictors with a statistically significant linear relationship for ^(*)^ p< 0.5 or ^(**)^ p< .20 in bold

## Discussion

The aim of the present study was to investigate, whether it is possible to predict the KSS scores based on PSF and SMF obtained during computer-assisted TKA. The approach used is new. Fifteen potentially relevant SMF were accessed, which in part cannot be measured by radiographic morphometry. To the authors’ best knowledge there are no publications available addressing such a high number of SMF. Neither are there any publications available addressing the relationships between PSF, SMF and the KSS ratings.

The key findings of the present study are: (1) PSF could explain 11 % of the 4-years KSS-F scores variability; (2) four SMF could explain 18 % of the 4-years KSS-F variability, however, the unstandardized coefficients (B) of two of the factors considered in the model (FJCmin and FGDM-L) could not be explained, therefore these factors were excluded from the final model; (3) a combination of PSF and SMF could explain the largest proportion (20 %) of the 4-years KSS-F variation.

According to the present results, older age, higher BMI and lower preoperative KSS-F ratings are preconditions that negatively influence the 4-years KSS-Function outcomes.

Several meta-analyses have shown that navigated TKA significantly improves prosthesis alignment, component position and limb alignment [34–36]. Only a few reports have shown that navigation can result in improved functional outcomes in TKA [21, 37].

In a meta-analysis investigating the influence of BMI on the outcomes of primary TKA a trend was reported for a lower postoperative KSS in obese (BMI ≥ 30 kg/m^2^) patients than in non-obese (BMI < 30 kg/m^2^) patients [38]. The present results reinforce this finding.

There is some evidence supporting the hypothesis that SMF, like the PTS and the PCO, influence the maximal knee flexion (MKF) after TKA [14, 39–43]. Since knee flexion is also assessed by the KSS and influences the performance of functional activities, it was hypothesized that these variables could probably predict 4-years KSS-F. According to the expectations and reinforcing the above mentioned studies, the “Tibial Component Slope” (TCS) and the “Femoral Offset Changes Medial” (FOCmed) were considered in the final model, as predictors of the 4-years KSS-F. Higher positive TCS values (which represents the same as higher posterior TCS values) were significant predictors of a higher 4-years KSS-F score. Small or no changes in FOC (the same as PCO), were also significant predictors of better 4-years KSS-F scores. The present results support the ones of the above referred studies.

For the present sample the strongest predictors of 4-years KSS-F were the TCS, followed by BMI and Age. The present results support early knee replacement surgery once “Age” was significantly negatively correlated with 4-years KSS-F scores. Furthermore the “Pre-OP KSS-F” score was positively correlated with “4-years KSS-F” (Table 5).

Surprisingly, no significant predictors of KSS-K score were found, neither among SMF, nor among PSF. KSS-K is the more objective KSS score, since it is based on the assessor measurements. The authors have no explanation for this finding. Maybe the conversion of the more objective data into to the KSS-K score, with the use an algorithm, is behind this findings.

Due to the fact that this study is a secondary analysis of data, a prospectively performed power analysis wasn’t possible. This is a study limitation. Further studies on this issue should be prospectively statistically powered. A second study limitation is related to the fact that co-morbidities were not accessed by the time the patients entered the study. Co-morbidities may significantly predict the results and should be accessed in further studies. A third study limitation concerns the fact that navigation data were obtained based on measurements upon the resected bone surfaces and further calculated by the software of the OrthoPilot system. Future studies on this matter should consider additional measurements after final implantation of the components, since the depth of cement mantle may influence the implant position and consequently the measured gaps.

The results of studies in this area could be used to identify patients at risk of poor outcomes submitting for TKA, as suggested by Lungu et al. [44]. The use of a regression equation prior to surgery to identify patients at risk could help to choose the appropriate course, such as pre-rehabilitation, participation in a weight-loss program before surgery or intensive post-operative rehabilitation.

## Conclusions

Computer-navigation is a suitable tool to accurately measure and control a variety of SMF, which seem to affect clinical outcomes after TKA. Two PSF alone accounted for 11 % of the 4-years KSS-F variation. A combination of three PSF and two SMF explained 20 % of the 4-years KSS-F variability. According to the results of the present study, younger age, better preoperative KSS-F scores and lower BMI before surgery, a positive (posterior) Tibial Component Slope (TCS) and small changes in Femoral Offset (FOC) were predictors of better KSS-F scores at 4-years after TKA.

## Abbreviations

BMI: body mass index
FB: fixed bearing
HRQL: health related quality of life
KSS: knee society score
KSS-F: knee society function score
MB: mobile bearing
Pre-OP MKF: pre-operatively maximal knee flexion
OA: osteoarthritis
PCO: posterior condylar offset
Pre-OP KSS: pre-operatively knee society score
PSF: patient specific factors
PTS: posterior tibial slope
ROM: range of motion
SMF: surgically modifiable factors (see also Table 2)
TKA: total knee arthroplasty
